# Second Primary Malignancies Post Autologous Stem-cell Transplant in Multiple Myeloma from India – Knowing the Unknown

**DOI:** 10.1007/s12288-025-01984-2

**Published:** 2025-04-26

**Authors:** Sumeet Mirgh, Athira Surendran, Sachin Punatar, Anant Gokarn, Nishant Jindal, Akanksha Chichra, Lingaraj Nayak, Prashant Tembhare, Nikhil Patkar, Sweta Rajpal, Gaurav Chatterjee, Libin Mathew, Papagudi Subramanian, Bhausaheb Bagal, Navin Khattry

**Affiliations:** 1 BMT Unit, Department of Medical Oncology, Tata Memorial Centre, ACTREC, Kharghar, Navi Mumbai, 410210 India; 2 Homi Bhabha National Institute (HBNI), Anushakti Nagar, Mumbai, India; 3 Department of Hematopathology, Tata Memorial Centre, ACTREC, Kharghar, Navi Mumbai, 410210 India

**Keywords:** Multiple myeloma, Autologous stem cell transplant, Second malignancy, India

## Abstract

To evaluate the incidence, clinical characteristics and outcomes of second primary malignancies (SPM) in Multiple myeloma patients post autologous stem-cell transplant (ASCT). This is a report of myeloma patients from a prospectively maintained database who underwent ASCT between 1st March, 2007 to 31st December, 2022. Baseline characteristics, details of induction, transplant and post-transplant maintenance therapy of these patients were retrieved. Details of treatment received for SPM, and outcomes were also obtained. In total,178 patients underwent 192 ASCTs (14 patients with two ASCTs). Of 178 patients, six patients (3.3%) developed SPM [3-solid (1.6%) and 3-haematological (1.6%)] at a median follow-up post-ASCT of 8.7 years. Median follow-up of entire cohort (*n* = 178) post-ASCT was 51 months. Median time from diagnosis and transplant to second malignancy was 9.0 years (5.4–13.5 years) and 8.2 years (4.0-12.3 years), respectively. Median duration of lenalidomide exposure from diagnosis to second malignancy in these six patients was 25.8 months (Range: 15–40 months). Amongst our six patients with SPM, three patients (50%) are alive. At a median follow-up of more than 8 years post-ASCT, the incidence of SPM was 3.3% in our cohort. This highlights the need for life-long surveillance for SPM in myeloma patients post-ASCT.

## Introduction

In newly diagnosed transplant eligible multiple myeloma (MM) patients, Triplet (Bortezomib-lenalidomide-dexamethasone - VRd) [1, 2] or quadruplet (Daratumumab + VRd) [3] regimens followed by autologous stem-cell transplant (ASCT) and lenalidomide maintenance is standard of care treatment. Consolidation with ASCT is important for long-term disease control. One long-term complication of ASCT is second primary malignancy (SPM), which may possibly be compounded by use of novel agents like lenalidomide. A large retrospective analysis over two decades from Japan suggests, that the incidence of second primary malignancy (SPM) has increased in the era of novel agents compared to pre-novel agent era (4.3% vs. 1.9% at 5-years) [4]. However, whether this is due to increased life-expectancy consequent to better disease control with novel agents is debatable. In patients with MM, the risk of developing SPM varies between 3.5 and 6.9% [5]. As compared to age-matched control population, this risk is higher for MM patients, especially for hematological malignancies [6]. Data from a large California-cancer registry (*n* = 16,331 MM patients) showed that 10-year cumulative incidence of a second “hematological SPM” is higher in ASCT recipients [7]. However, 10-year cumulative incidence of developing any “invasive SPM” is similar in transplant and non-transplant patients [1, 2, 7].

In India, MM accounts for 1.2% of all cancers, with an incidence of 1 per 100,000 population [8]. ASCT for MM accounts for 20% of all transplants, and 50% of all autologous transplants done in our country [9]. However, there is paucity of data from India which highlights the risk of SPM post-ASCT in our population.

## Methods

This is a retrospective single-centre analysis of MM patients (*n* = 178) who underwent ASCT between 1st March, 2007 to 31st December, 2022. The patients with SPM were identified from a prospectively maintained database. Patients with a diagnosis of another malignancy prior to or concomitant with MM diagnosis were excluded.

Patients aged 18–65 years of age with a diagnosis of MM received a doublet (before year 2011) or triplet-induction regimen (Bortezomib + dexamethasone + lenalidomide or Thalidomide or Cyclophosphamide), followed by stem cell (PBSC) collection after achieving ≥ PR (Partial response). PBSC were mobilised using either chemo-mobilization or G-CSF + Plerixafor, and cryopreserved as reported before [10]. Patients received Melphalan (140-200mg/m^2^) conditioning followed by PBSC infusion after 24 h. Post-transplant, patients were followed up for 3–4 months with serum protein electrophoresis, serum immunofixation and serum free-light chain assay with ratio.

For patients with SPM, patient characteristics (age at diagnosis, ASCT and diagnosis of SPM; gender, comorbidities), disease-related details (date of diagnosis, ISS and R-ISS staging, baseline bone marrow morphology, baseline extramedullary disease), treatment characteristics (frontline induction, receipt and site of radiotherapy, duration of lenalidomide and response prior to ASCT), transplant-related details [mobilization regimen for ASCT, stem-cell dose collected and infused, date of ASCT, melphalan dose, date of second ASCT (if any), post-ASCT maintenance and duration, marrow findings and PET-CT at relapse, salvage regimen for relapse] and SPM related details [date of diagnosis, site of SPM, type (solid or hematological), treatment, status at last follow-up] were captured. Additionally, cumulative duration of immunomodulatory drugs, lenalidomide, and cyclophosphamide (in months) were also recorded. Cut-off date for last follow-up was kept as 31st March, 2024.

SPM was defined as an event, whenever a patient was diagnosed as a second malignancy (except a plasma cell dyscrasia), which was proven histologically or by flow-cytometry. Both invasive and non-invasive second cancers were counted in the SPM group. Overall survival was calculated from date of ASCT (date of first ASCT for patients with two ASCTs) to date of death, from any cause. Progression-free survival was defined as time from ASCT (date of first ASCT for patients with two ASCTs) to (IMWG defined) relapse or progression of MM or death from any cause. The cumulative incidence of SPM was calculated from time of ASCT (date of first ASCT for patients with two ASCTs), with death (not due to SPM) as a competing event. All P-values were 2-sided tests, with a value 0.05 considered as significant.

## Results

Between March 2007 – December 2022, a total of 178 MM patients underwent 192 ASCTs (14 patients underwent two ASCTs). Of these 178 patients, six patients (3.3%) developed SPM [solid (*n* = 3;1.6%) and haematological (*n* = 3;1.6%)] at a median follow-up post-ASCT of 8.7 years **(**Table 1**)**. Median follow-up of entire cohort (*n* = 178) post-ASCT was 4.3 years. Most common isotype at diagnosis was IgG Kappa [*n* = 5;83.3%]. ISS distribution in the six patients were – ISS-I (*n* = 3;50%), ISS-II (*n* = 2;33%), and ISS-III (*n* = 1;16%). Baseline cytogenetics by FISH were available in three patients, of which two were standard risk. Frontline induction was a doublet in 4 patients, and triplet in remaining two patients. Lenalidomide was used as a part of induction regimen in four patients (66%). For stem-cell apheresis, five patients underwent chemo-mobilization (*n* = 4 with Cyclophosphamide + G-CSF; *n* = 1 with Bortezomib + G-CSF), while one received G-CSF + Plerixafor. Four patients were in ≥ VGPR prior to ASCT. One patient underwent tandem ASCT. Median CD34 stem cell-dose infused was 3.34 × 10^6^/kg.

**Table 1:** Comparison of characteristics of patients with and without second primary malignancies (SPM)

	Without SPM (*n* = 172)	With SPM (*n* = 6)	*P* value
Median age (years)	41.5	51	0.72 (NS)
Gender [Male: Female]; n (%)	124:48 (72%)	2:4 (33%)	0.11 (NS)
Melphalan dose in 1st ASCT (mg/m^2^)			
Mel 200 Mel < 200	128 (75%) 44 (25%)	5 (83%) 1 (16%)	0.98
Induction regimen			
Doublet Triplet	22 (13%) 150 (87%)	4 (66%) 2 (33%)	0.0002
Doublet induction *			
Rd Td Vd Others	4 (2%) 9 (5%) 8 (5%) 1 (0.5%)	3 (50%) 1 (16%) 0 0	0.15
Triplet induction *			
VCd VRd VTd Others	86 (50%) 64 (37%) 15 (9%) 1 (0.5%)	1 (16%) 1 (16%) 0 0	0.95 ^#^
Receipt of lenalidomide in induction; n (%)	80 (46%)	4 (66%)	0.57
Receipt of radiation therapy during treatment; n (%)	62 (36%)	1 (16%)	0.58
Number of patients with two transplants N (%)	13 (7.6%)	1 (16%)	0.96
Median follow-up from ASCT-1 (months)	52.13	106.9	0.009
Deaths; n (%)	52/172 (30%)	3/6 (50%)	0.56

*Total number of patients with doublet and triplet induction regimens may exceed the actual number of patients for first column (patients without SPM), as some patients have received two lines of induction treatment prior to transplant, in view of inadequate response; # compared for VCd vs. other triplet inductions

With respect to SPM, median time to diagnosis of second malignancy was 9.0 years (Range: 5.4–13.5 years) and 8.2 years (Range: 4.0-12.3 years) after diagnosis of myeloma and ASCT, respectively. Median time from transplant to diagnosis of hematological SPMs was 7.2 years (Range: 4.3–12.1 years). Amongst haematological malignancies **(**Table 2**)**, two patients developed AML (one with complex karyotype and one with monosomal karyotype). Both developed AML as a late complication (7 years, and 11.7 years post-ASCT). Third patient developed therapy-related B-ALL at 4 years post-ASCT. He had hyperdiploid karyotype, and achieved MRD negative complete remission (CR) after treatment with a pediatric-inspired modified BFM90 protocol. Importantly, he developed a life-threatening pneumonia during his phase 1a induction with transfusion-dependant cytopenias lasting 25 days. In view of his persistent cytopenias, and diagnosis of two malignancies, we performed stress cytogenetics from peripheral blood, which was negative. He is currently alive 6 months after diagnosis, and continues to be in CR, for both his plasma cell dyscrasia, and B-ALL.

**Table 2:** Details of myeloma and second primary malignancies

Sr No	Age at BMT	Gender	Myeloma cytogenetics	Lenalidomide in induction	Melphalan dose (mg/m^2^)	Maintenance therapy drug	SPM and its characteristics	Time from BMT to diagnosis of SPM (years)	Treatment	Outcome
1	53	F	NA	Yes	200	Lenalidomide	Papillary carcinoma of thyroid (less than 1 cm)	12.3	Observation	Surviving at 4 months after SPM diagnosis
2	47	F	NA	Yes	200	No	MDS with complex karyotype del(20q); del(7q); Monosomy 5; Trisomy 8	7.0	Supportive care alone	Succumbed to her SPM at 3 months after diagnosis
3	45	F	14q del; 1q amp; 1p del	No	200	Bortezomib	Periampullary carcinoma pT3N0	6.0	PPPD f/b 6 cycles of adjuvant Gemcitabine	Surviving at 1 year 9 months after SPM diagnosis
4	57	M	NA	No	180 (ASCT1) 200 (ASCT2)	No No	Gastric carcinoma Details unknown	9.5	Not known	Succumbed to his SPM at 2 months after diagnosis
5	35	M	Trisomy 3,5,7,9; t(14;?); Monosomy 13	Yes	200	Lenalidomide	B-ALL; Cytogenetics - tetrapentasomy 10 and 14, tritetrasomy 1,4,10,19; NGS - negative	4.0	mBFM90 ongoing	Surviving at 4 months post SPM diagnosis and undergoing treatment. Achieved CR with flow-MRD negative post phase 1 induction
6	49	F	Trisomy tetrasomy 11	Yes	200	Lenalidomide	AML; Cytogenetics - del5q and del7q; NGS - TP53; DNMT3A; RAS mutation	11.7	Not known	Succumbed to her SPM at 2 months after diagnosis

Abbreviations - ASCT1,autologous stem cell transplant 1; ASCT2,autologous stem cell transplant 2; amp, amplification; CTG, cytogenetics; del, deletion; F, female; M, male; mBFM90, Modified BFM90 protocol; MRD, Minimal residual disease; NGS – Next Generation sequencing; PPPD, pylorus preserving pancreatico-duodenectomy; SPM, second primary malignancy

Three patients developed solid tumors **(**Table 2**)** at a median duration of 9.5 years (Range: 6-12.5 years) post-ASCT. The types of solid tumors were varied – one patient each developing papillary carcinoma of thyroid (PTC), periampullary pancreatic cancer, and gastric carcinoma. The patient with PTC was kept on observation as the thyroid nodule size was sub-centimetre size, and she is alive at 4 months after diagnosis of SPM. Meanwhile, the patient with pancreatic cancer was staged pT3N0, and he underwent curative Pylorus-Preserving-Pancreato-duodenectomy (PPPD) followed by 6 cycles of adjuvant gemcitabine. The third patient had a metastatic gastric cancer, was put on palliation and he succumbed to his SPM after one month.

Median time from diagnosis of MM to diagnosis of SPM was 8.1 years (Range: 5.8–13.1 years) vs. 10.4 years (Range 8.9–13.2 years) for hematological vs. solid SPMs, respectively. All six patients received lenalidomide, either during their initial line of treatment or at relapse, prior to development of SPM. Median duration of lenalidomide exposure from diagnosis of myeloma to SPM in these six patients was 25.8 months (Range: 15–40 months). Only one patient received palliative radiotherapy to D3-D5 region (in view of wedge compression fracture causing cord compression). She developed pancreatic cancer 86 months after radiotherapy.

**Fig. 1:**
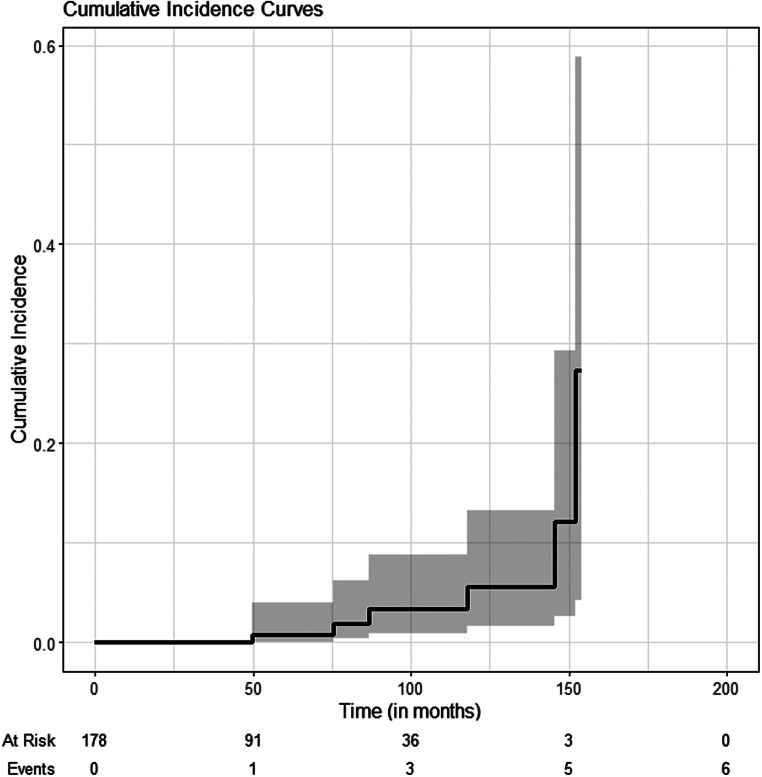
Cumulative incidence of second primary malignancy in our cohort

Median age of patients with and without SPM was 41.5 years and 51 years (*p* = 0.72), respectively. Amongst 14 patients who underwent two ASCTs, only one (7%) developed an SPM. None of our patients had a relevant family history of malignancies. Next generation sequencing was done in two patients (Patient 5 and 6 in Table 2) which did not reveal any germline mutation. Median follow-up from time of ASCT was 107 months vs. 52 months in patients with and without SPM (*p* = 0.009), respectively. Numerically, deaths were higher in patients with SPM (50%), vs. those without SPM (30%) (*p* = 0.56). Amongst patients without SPM (*n* = 172), most common cause of death was relapse (*n* = 40;77%). Cumulative incidence of SPM in our entire cohort (*n* = 178) at 5 years and 10 years post-ASCT was 0.8% (95% CI – 0.07-4%) and 5.6% (95% CI – 1.6-13%), respectively **(**Fig. 1**)**.

## Discussion

With increasing survival in MM, with the use of novel agents, there is also an increased risk of SPM. We performed a single-centre retrospective study in MM patients covering a 15-year period to highlight the risk of SPM post-ASCT.

Our SPM incidence of 3.3% at a median follow-up of 8.7 years post-ASCT, is similar to reported literature (2.5-4%) [4, 5, 11]. DETERMINATION trial showed us that the incidence of SPM in patients who underwent ASCT vs. those without was similar (10.7% vs. 10.4%) at a median follow-up of 76 months [2]. Importantly, randomised studies of transplant vs. no-transplant have highlighted that the cumulative incidence (CI) of hematological SPM was higher in ASCT recipients as per IFM trial (5-year CI 1.4% vs. 0.6%) [1], DETERMINATION trial (5-year CI 3.5% vs. 1.6%) [2], and UK NCRI trial (7-year CI 2.8% vs. 1.4%) [12]. Higher incidence in DETERMINATION trial [2] as compared to IFM [1] underlines the fact that possibly there is an increased risk of hematological SPM with indefinite duration of lenalidomide. The higher CI of hematological SPM in transplant arms is much higher than reported in non-transplant studies [13], which used indefinite duration of lenalidomide (0.5%). Our incidence of hematological SPM (1.6%) is lesser, as compared to that reported in DETERMINATION trial (3.6%) [2], probably because all patients (46%) did not receive lenalidomide as induction regimen in our cohort. Samur et al. showed that high dose melphalan increased the mutational burden at relapse post-ASCT in patients with MM [14]. But, how these newly acquired mutations associated with DNA damage clinically influence the possibility of developing an SPM is unclear. Similar to literature from India [15], only 8% of our patients underwent two transplants. However, in such patients who received higher cumulative dose of melphalan, the risk of SPM did not appear to be increased **(**Table 1**)**.

Similar to Spanish data [16], we also showed that outcomes of MM patients who develop therapy-related myeloid neoplasms (t-MNs) are poor, with majority of them having a complex karyotype, and a median OS of 4 months. A study from Mayo clinic highlighted that abnormal immunophenotype of myeloid progenitor cells and immune effector cells pre-transplant and pre-maintenance served as an early predisposition to t-MNs in MM patients post-ASCT [17]. Similar to literature [5], the constellation of solid SPMs post-ASCT in our cohort was heterogenous. The outcome of solid SPM post-ASCT is debatable [5], with some studies reporting their outcomes similar to denovo patients [18]. In our cohort, deaths were numerically higher in those who developed SPM vs. others, similar to CIBMTR analysis [10]. However, the number of patients in our SPM cohort is less to derive conclusions. The median follow-up post-ASCT was higher in our patients who developed SPM vs. those without (107 months vs. 52 months). This re-iterates the fact that development of SPM is an event which increases with time.

There is paucity of Indian data which reports the incidence of SPM post-ASCT in MM. Kumar et al. reported long-term results of transplant in MM. In their cohort of 207 patients with a median follow-up of 7.5 years, 3(1.4%) developed an SPM. While 56% of their patients received novel-agent based induction, the number of patients who received lenalidomide have not been specified. Importantly, they included patients from 1990 to 2013. Lenalidomide maintenance was given to patients in their cohort after 2010. This implies that only a small number of patients must have received lenalidomide maintenance [19]. In another recent report from the same group, 1.5% (*n* = 7/459) patients developed SPM at a median follow-up of 88 months [20]. In our entire cohort, 48% (*n* = 87 out of 178) received cyclophosphamide as part of induction **(**Table 1**)**, and 70% received it as part of chemo-mobilization [21] (S.M., et al. Manuscript under review). Whether increased use of cyclophosphamide in our cohort, led to more SPM than reported by Kumar et al. (3.3% vs. 1.5%), remains speculative. To our knowledge, ours is the second study from India which has highlighted the incidence of SPM post-ASCT.

To conclude, our study re-iterates the need for continued surveillance for development of SPM post-ASCT in MM patients.
